# Consumers’ attitudes toward 3D food printing: A South African context

**DOI:** 10.1111/1750-3841.70198

**Published:** 2025-04-09

**Authors:** Nicole Cunningham, Adeyemi Ayotunde Adeyanju, Oluwafemi Ayodeji Adebo

**Affiliations:** ^1^ Department of Marketing Management, College of Business and Economics University of Johannesburg Johannesburg South Africa; ^2^ Centre for Innovative Food Research (CIFR), Department of Biotechnology and Food Technology, Faculty of Science University of Johannesburg Johannesburg South Africa

**Keywords:** 3D‐printed food, attitude, convenience, food neophobia, knowledge awareness

## Abstract

**Abstract:**

Three‐dimensional food printing is an emerging technology for processing food that could contribute to the goal of feeding about eight billion people in 2030. Considering the gradual uptake of this technology in other parts of the world and potentially in Africa, there is a need to understand consumers’ attitudes toward this technology and subsequent products to provide evidence that might guide business decisions among others. This study adopted an online survey from which 355 usable responses were received and subsequently analyzed. Using partial least squares structural equation modeling, the study proves that knowledge awareness, experience awareness, benefits, and health positively influence attitudes toward three‐dimensional‐printed food. The benefits associated with three‐dimensional‐printed food reveal the strongest relationship with attitude. However, food familiarity and convenience do not have a significant impact on attitude, signaling that consumers’ familiarity with three‐dimensional‐printed food could be related to their perceptions of convenience. Moreover, the study shows that food neophobia has a negative yet significant impact on attitude, signifying the importance of managing the levels of food neophobia. The study contributes to the understanding of consumers’ attitudes within a region where three‐dimensional food printing is still in its infancy. However, given its potential positive impact, particularly in Africa, understanding and affecting the attitudes toward three‐dimensional printed food is significant.

**Practical Application:**

This research will assist developers and marketers in understanding consumer attitudes toward 3D‐printed food. Food neophobia (the fear of trying new food) significantly affects the consumer's attitude negatively, while the benefits that 3D‐printed food offers present the strongest positive influence on attitude. The study entrenches the need for developers and marketers of 3D‐printed food to highlight the benefits that 3D‐printed food offers to positively shape consumers’ attitudes.

## 1 INTRODUCTION

Three‐dimensional (3D) printing is an additive manufacturing technique used for various applications in healthcare, construction, and, more recently, in the food industry (Brunner et al., 2018). Its potential in the food industry is far‐reaching, as it can be used for valorization, converting underutilized food crops to innovative products, personalization (Mudau & Adebo, 2024), and long‐term crewed spaceflights (Zhang et al., 2022). This technique has also been employed in reducing food refusal and improving nutrition by creating visually and texturally appealing products (Smith et al., 2022) and meeting the needs of consumers, such as dysphagia patients (Molimi et al., 2025).

This innovative technology holds significant potential in South Africa and the African continent as a whole, where food insecurity remains a serious concern, with many communities struggling to access affordable, nutritious, and diverse food options (Van der Berg et al., 2022). Traditional food production methods are resource‐intensive and often fall short of meeting the needs of a growing population, especially in low‐income areas. 3D food printing offers a unique solution by enabling the creation of nutrient‐dense, customized foods tailored to specific dietary needs. It also supports sustainability by incorporating alternative ingredients, reducing food waste, and optimizing resource use, thereby potentially improving food accessibility and affordability for South African communities. However, despite the numerous benefits of 3D food printing, the role of consumers in the food chain cannot be overemphasized.

Food neophobia, which relates to the fear of consuming new food, is of concern and a worldwide phenomenon (Lee et al., 2021; Pliner & Hobden, 1992). 3D‐printed food neophobia has been associated with the fear of new technology and relates to the “unnatural” state of the process, sanitation, and safety during printing (Brunner et al., 2018; Lupton & Turner, 2018; Manstan et al., 2021). This was buttressed by Ross et al. (2022), who indicated that consumer acceptance of 3D food printing as a new food processing technology can be somewhat challenging due to unfamiliarity to potential consumers. Kocaman et al. (2023) suggested that future research should focus on how the human–food relationship is influenced by 3D‐printed food. Therefore, it is imperative to study consumers’ attitudes to 3D‐printed food and understand the acceptability of this product and process technology in Africa.

Attitudes, understood as a psychological tendency to evaluate a specific entity positively or negatively, are essential for understanding consumer acceptance of new technologies (Ajzen, 2001). Positive attitudes toward 3D food printing are often driven by an appreciation for the technology's novelty and potential to address modern food challenges, while negative attitudes are frequently tied to skepticism about its healthiness and authenticity (Lupton & Turner, 2018). Examining these attitudes is essential for understanding consumer behavior and adoption patterns, as well as for identifying potential market barriers and strategies for fostering acceptance. Globally, consumer attitudes toward 3D‐printed food have shown diversity, shaped by factors including familiarity with the technology, perceived advantages, cultural acceptance, and awareness levels (Brunner et al., 2018; Caulier et al., 2020; Ng et al., 2022). Additionally, the type of 3D‐printed food plays a significant role in shaping consumer attitudes, as different food types elicit varying levels of acceptance and interest. Research indicates that consumers are generally more receptive to 3D‐printed plant‐based foods because of their perceived health benefits and lower environmental impact relative to meat products. In contrast, 3D‐printed meat or fish products may encounter more skepticism, often stemming from concerns about taste, authenticity, and naturalness (Lupton & Turner, 2018; Lanz et al., 2024).

Some research has emanated from other parts of the world on the use of 3D‐food printing for different products (i.e., Fasogbon & Adebo, 2022; Kewuyemi, Kesa, & Adebo, 2022; Zhang et al., 2022; Zhu et al., 2023) as well as an understanding of consumer acceptability, attitude, and perceptions (Caulier et al., 2020; Feng et al., 2022; Manstan & McSweeney, 2020; Manstan et al., 2021; Ross et al., 2022). However, only a few studies have emanated from Africa on 3D food printing for different products (Fasogbon & Adebo, 2022; Kewuyemi et al., 2023; Kewuyemi, Kesa, Meijboom, Alimi, & Adebo, 2022; Molimi et al., 2025) and, to the best of our knowledge, no studies have examined consumers’ attitudes toward 3D‐printed food. Considering some increased research and investment in this regard, it is pertinent to investigate this topic given the potential positive societal change and food security that 3D‐printed food can bring to Africa. Specific factors were considered that could influence attitudes toward 3D‐printed food products, particularly knowledge awareness, experience awareness, benefits, food familiarity, and food neophobia. Thus, this research aims to assess South African consumers’ acceptance, preferences, and concerns regarding 3D‐printed foods, identifying potential barriers and motivators that could influence adoption. Insights gained will guide the development of marketable, culturally appropriate, and nutritious 3D‐printed food options that align with local dietary needs and contribute to food security and sustainability.

## 2 RESEARCH METHODOLOGY

**FIGURE 1:**
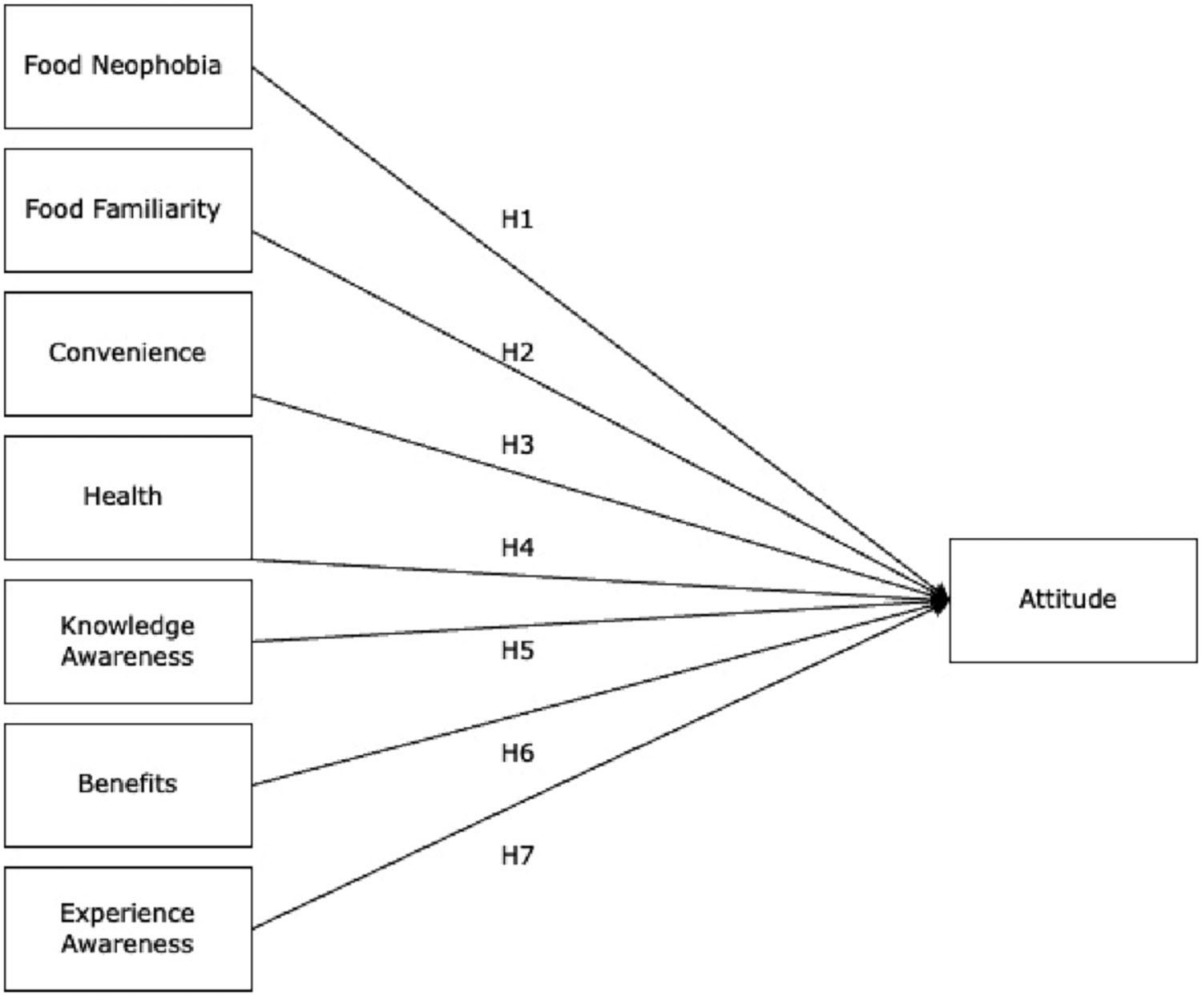
Conceptual model representing the relationships predicted to influence the attitude of 3D‐printed food tested in this study.

Ethical approval was requested for and received from the University of Johannesburg's Faculty of Science Ethics Committee, with approval reference 2023‐05‐03/Adebo. The study was quantitative in nature and followed a descripto‐explanatory research design, which, according to Saunders et al. (2019), allows for the detailed description and analysis of the variables under study and their associated relationships (as shown in Figure 1). The target population for this study included consumers in South Africa who were aged 18–65 and who were familiar with the concept of 3D‐printed food. Using purposive sampling, data was collected using an online questionnaire facilitated through Google Forms, which was shared on various social media platforms.

The self‐administered online questionnaire included a preamble outlining the objective of the study and the relevant ethical considerations. The questionnaire began with two screening questions (concerning the familiarity with 3D‐printed food as a concept and age) to ensure that the desired respondents participated in the study. The screening question focusing on whether the respondents were familiar with 3D‐printed food as a concept was necessary given the limited access to 3D‐printed food. This was followed by a section focusing on building a demographic profile (i.e., age, gender, race, level of education, employment status, income, and province of residence). The next section made use of a seven‐point Likert‐type scale, where 1 indicated “strongly disagree” and 7 indicated “strongly agree” to obtain responses to the eight constructs proposed in this study (refer to Figure 1). The eight constructs and their corresponding items are presented in Table 1. The knowledge awareness, experience awareness, benefits, food familiarity, food neophobia, and attitude constructs and their corresponding items used in the study were adapted from Ng et al. (2022), while convenience orientation and health were adapted from Brunner et al. (2018). In total, 355 usable responses were received and analyzed. Responses were deemed usable if respondents met the inclusion criteria (the two screening questions as mentioned above) and the questionnaire was complete.

**TABLE 1:** Reliability (Cronbach alpha and composite reliability) and convergent validity of each construct and its corresponding items.

Construct and items	Factor loadings	*α*	CR	AVE
Knowledge awareness				
I have already heard or read about 3D‐printed food.	0.876	0.795	0.905	0.826
I roughly know how the 3D printing process works.	0.941			
Experience awareness				
I have consumed 3D‐printed food before.	0.972	0.948	0.975	0.950
I have dealt with 3D‐printed food before.	0.978			
Benefits				
3D food printing can produce food easier and more conveniently.	0.717	0.911	0.928	0.618
3D food printing can produce food to meet the dietary requirements of people with health issues.	0.707			
3D food printing can design food with personalized nutritional needs.	0.731			
3D food printing can help reduce food wastage.	0.789			
I have no trouble consuming 3D‐printed food because it is safe to consume.	0.857			
I have no trouble consuming 3D‐printed food because it is beneficial to my health.	0.806			
I have no trouble consuming 3D‐printed food because it is a fun experience.	0.828			
I have no trouble consuming 3D‐printed food because it is not disgusting.	0.836			
Food familiarity				
It is important to me that the food to be eaten is usually what I would eat.	0.879	0.840	0.903	0.756
It is important to me that I am familiar with the food I would eat.	0.885			
It is important to me that the food I eat is similar to the food I ate during my childhood.	0.844			
Food neophobia				
I am afraid to eat food that I have never eaten before.	0.754	0.898	0.918	0.586
I will not try a new food if I do not know what is in it.	0.749			
I do not trust new food.	0.805			
I do not like trying out new and different food.	0.821			
I do not need new food technology because there is already much tasty food around.	0.807			
The benefits of new food technology are always overstated.	0.782			
New food technology reduces the natural quality of food.	0.590			
I will not try out high‐tech food products because the ones I eat are already good enough.	0.791			
Convenience orientation				
The less physical energy I need to prepare a meal, the better.	0.888	0.933	0.949	0.789
The ideal meal can be prepared with little effort.	0.907			
Preferably, I spend as little time as possible on meal preparation.	0.904			
I want to spend as little time as possible cooking.	0.890			
At home, I preferably eat meals that can be prepared quickly.	0.850			
Health				
It is important to me that the food I eat on a typical day contains a lot of vitamins and minerals.	0.864	0.945	0.956	0.784
It is important to me that the food I eat on a typical day keeps me healthy.	0.907			
It is important to me that the food I eat on a typical day is nutritious.	0.898			
It is important to me that the food I eat on a typical day is high in protein.	0.900			
It is important to me that the food I eat on a typical day is good for my skin, teeth, hair, nails, etc.	0.875			
It is important to me that the food I eat on a typical day is high in fiber and roughage.	0.869			
Attitude				
I think that 3D food printing is generally good.	0.897	0.922	0.945	0.811
I think that 3D food printing is generally important.	0.883			
I think that 3D food printing is generally to be supported.	0.913			
I think that 3D food printing is generally positive.	0.909			

Abbreviations: AVE, average variance extracted; CR, composite reliability; *α*, Cronbach's alpha value.

The following hypotheses were put forward in this study: Food neophobia, characterized by fear and resistance to trying new foods, negatively impacts attitudes toward 3D‐printed food (H1) (Lee et al., 2021). In contrast, familiarity with food and related technologies fosters positive attitudes by helping consumers visualize the technology's role in daily life (H2) (Lee et al., 2021; Ross et al., 2022). Convenience, a significant factor in food acceptance, positively influences attitudes toward 3D‐printed food due to its ability to simplify meal preparation (H3) (Brunner et al., 2018). Health‐conscious consumers are drawn to 3D‐printed food for its potential to personalize nutrition and improve access to healthier options (Manstan & McSweeney, 2020), which enhances positive perceptions (H4). Knowledge awareness about 3D‐printed food also positively affects attitudes, as greater understanding reduces skepticism (H5) (Caulier et al., 2020). Perceived benefits, including convenience, health, flavor, nutrition, and environmental advantages, encourage favorable attitudes when the benefits outweigh the costs (H6) (Chang et al., 2024; Lee et al., 2021; Manstan & McSweeney, 2020; Ross et al., 2022). Finally, personal experience with 3D‐printed food positively impacts attitudes, as familiarity breeds comfort and willingness for future consumption (H7) (Manstan et al., 2021).

Prior to testing the hypotheses, the threat of common‐method variance was assessed using Harman's single‐factor test. The unrotated factor solution showed that the single factor accounted for less than 50% of the total variance. Therefore, according to Podsakoff and Organ (1986), the data were free from common‐method bias. Using SmartPLS version 4, the analysis consisted of the analysis of the reliability (Cronbach's alpha and composite reliability [CR] coefficients) and validity (convergent and discriminant validity), whereafter the hypotheses were assessed by reviewing the path estimates and their level of significance.

## 3 RESULTS

### 3.1 Reliability and validity

The Cronbach's alpha values for various constructs were: 0.795 for knowledge awareness, 0.948 for experience awareness, 0.911 for benefits, 0.840 for food familiarity, 0.898 for food neophobia, 0.933 for convenience orientation, 0.945 for health, and 0.922 for attitude. The results suggested a generally positive reception, supported by strong internal consistency across various constructs. Specifically, the high Cronbach's alpha values indicated reliable measures for knowledge awareness, experience awareness, benefits, food familiarity, food neophobia, convenience orientation, health considerations, and attitude. A Cronbach's alpha value above 0.7 typically signifies good internal consistency, which in this case indicates that the construct items reliably captured the constructs they intended to measure (Narayanamurthy & Tortorella, 2021). Consequently, these findings implied that the respondents had a significant level of understanding, positive experiences, perceived benefits, and favorable attitudes toward 3D food printing and its products.

The high CR values obtained for the various constructs in the study on consumers’ attitudes toward 3D food printing and 3D‐printed products also suggested robust internal consistency within the measurement model, as they met the 0.70 threshold. The CR values ranging from 0.903 to 0.975 indicated strong reliability and consistency in measuring the latent constructs of knowledge awareness, experience awareness, benefits, food familiarity, food neophobia, convenience orientation, health, and attitude. This implies that the survey items designed to capture these constructs were reliable and consistent in measuring the intended concepts. Such high reliability is crucial for ensuring that the findings accurately reflect consumers’ attitudes toward 3D food printing and its associated products, providing a solid foundation for drawing meaningful conclusions and making informed decisions based on the study's results. Furthermore, the high‐reliability values indicated a high level of confidence in the validity of the research instrument and the constructs it aimed to measure, enhancing the overall credibility and trustworthiness of the study findings.

The results of the assessment of convergent validity, which included the assessment of the factor loadings and the average variance extracted (AVE), are provided in Table 1. The results revealed that the factor loadings and the AVE values were above the 0.50 threshold (Fornell & Larcker, 1981). Therefore, the criteria for validity were met. The factor loadings and the AVE provided valuable insights into the relationships between the variables and the reliability of the measurement model. High factor loadings, such as those for knowledge awareness, experience awareness, benefits, food familiarity, convenience orientation, health, and attitude, indicated strong relationships between these constructs and the various items/indicators used for their measurement. This suggests that these constructs were reliably measured by the respective indicators (Gurer et al., 2019). Moreover, high AVE values for most constructs suggested that a large proportion of the variance in each construct was captured by its corresponding indicators, suggesting good convergent validity. However, slightly lower AVE values for food neophobia and benefits suggested that these constructs may be less well‐defined or measured by the indicators used in the study. Overall, these findings implied that consumers’ attitudes toward 3D food printing and 3D‐printed products are influenced by factors, such as knowledge, experience, perceived benefits, familiarity with 3D printed food, convenience, and health considerations, highlighting the importance of these factors in shaping consumers’ perceptions and acceptance of this emerging technology.

The heterotrait‐monotrait (HTMT) method of assessing discriminant validity was used in this study. As shown in Table 2, the results confirm discriminant validity. Assessing discriminant validity is essential in studies that involve latent variables and multiple items or indicators representing a construct. This step is necessary to confirm that the latent constructs used to measure causal relationships are truly distinct from each other. Ensuring discriminant validity helps to prevent the constructs from overlapping, thereby avoiding problems like multicollinearity (Ab Hamid et al., 2017; Henseler et al., 2015). It is usually assessed by checking if the correlations between constructs are smaller than the square roots of the AVE of each construct (Table 1). If the correlations are significantly smaller than the square roots of their respective AVEs, it indicates good discriminant validity, suggesting that the constructs are distinct from each other. The data obtained using the HTMT method suggested that the constructs demonstrated good discriminant validity.

**TABLE 2:** Discriminant validity using the HTMT method.

	ATT	BEN	CON	EXP	FAM	FN	H	KA
ATT		0.734	0.215	0.396	0.155	0.438	0.287	0.445
BEN	0.734		0.193	0.303	0.111	0.437	0.183	0.392
CON	0.215	0.193		0.080	0.148	0.234	0.527	0.401
EXP	0.396	0.303	0.080		0.035	0.241	0.045	0.213
FAM	0.155	0.111	0.148	0.035		0.544	0.115	0.140
FN	0.438	0.437	0.234	0.241	0.544		0.132	0.152
H	0.287	0.183	0.527	0.045	0.115	0.132		0.556
KA	0.445	0.392	0.401	0.213	0.140	0.152	0.556	

*Note*: Discriminant validity assesses whether a construct is truly distinct from other constructs—assessing the discriminant validity confirms that the constructs in the study measure different concepts. The HTMT method was used for determining discriminant validity by assessing if the correlations between constructs are smaller than the square roots of the AVE of each construct (Table 1). If the correlations are significantly smaller than the square roots of their respective AVEs, it indicates good discriminant validity, suggesting that the constructs are distinct from each other. The data obtained using the HTMT method suggested that the constructs demonstrated good discriminant validity.

Abbreviations: ATT, attitude; BEN, benefits; CON, convenience; EXP, experience awareness; FAM, familiarity; FN, food neophobia; H, habits; KA, knowledge awareness.

For example, the AVE value for attitude was 0.811, and its square root was 0.900. Similarly, the AVE for benefits was 0.618, and its square root was 0.786. The correlation between attitude and benefits (0.734) was less than both 0.900 and 0.786, indicating that discriminant validity was maintained between these two constructs. Generally, if the correlations between constructs are below the threshold of 0.85, it indicates distinctiveness between them (Ab Hamid et al., 2017). For instance, the highest correlation was between attitude and benefits (0.734), which was well below the 0.85 threshold, affirming that these constructs were sufficiently distinct (Table 2). Similarly, other correlations, such as between attitude and convenience orientation (0.215) or health and experience awareness (0.045), were much lower, further supporting discriminant validity. Therefore, these findings collectively indicated that the constructs measured in this study on consumers’ attitudes toward 3D food printing and 3D‐printed products were both valid and distinct, enabling accurate interpretation of consumers’ perceptions and attitudes.

### 3.2 Hypotheses analysis

**FIGURE 2:**
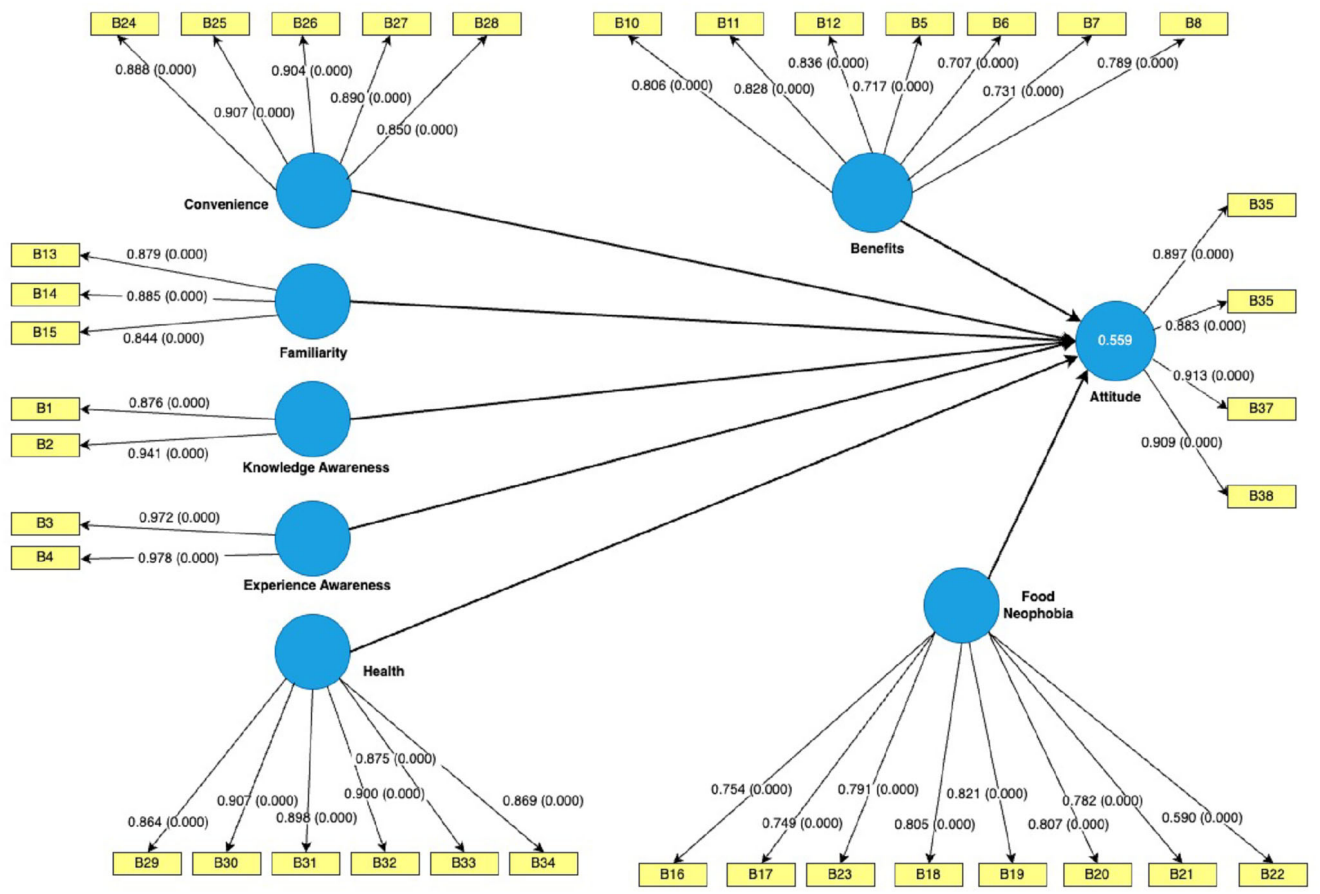
Smart PLS validated model showing the relationship, measurement, and structural components and assessment of the relationships toward the attitude of 3D‐printed food.

The results showing the main effects of the constructs are shown in Table 3 and Figure 2. As per Figure 2, the predictive ability (*R* ^2^) is 0.559. Table 3 presents the path coefficients and their level of significance. The results revealed that health (*β* = 0.109, *p* = 0.026), knowledge awareness (*β* = 0.103, *p* = 0.035), experience awareness (*β* = 0.162, *p* = 0.000), and benefits (*β* = 0.494, *p* = 0.000), had a positive and significant impact on attitude, providing support for H_4_, H_5_, H_6_, and H_7_. Food neophobia also had a negative but significant impact on attitude (*β* = −0.183, *p* = 0.000), providing support for H_1_. However, food familiarity had a negative yet significant impact on attitude (*β* = −0.004, *p* = 0.935), resulting in H_2_ not being supported, while convenience orientation had a positive yet negative impact on attitude (*β* = 0.075, *p* = 0.179), resulting in H_3_ being rejected.

**TABLE 3:** Results of the hypotheses testing.

Path	Estimate	*t*‐value	*p*‐value	Decision
Knowledge awareness → attitude	0.103	0.035	0.035	Supported
Experience awareness → attitude	0.162	4.118	0.000	Supported
Benefits → attitude	0.494	9.920	0.000	Supported
Food familiarity → attitude	−0.004	0.935	0.935	Not supported
Food neophobia → attitude	−0.183	3.667	0.000	Supported
Convenience orientation → attitude	0.075	1.345	0.179	Not supported
Health → attitude	0.109	2.223	0.026	Supported

*Note*: Estimate—refers to the coefficient of the independent variable and represents the predicted effect of the relationship (e.g., the effect of knowledge awareness on attitude); *t*‐value (test statistic)—refers to how many standard deviations the estimate is from zero to determine if its significantly different from zero (a larger *t*‐value means a stronger effect as its further from zero); *p*‐value (probability value)—indicates the probability of obtaining the observed estimate if the null hypothesis were true and helps determine if the effect (the estimate) is statistically significant. This helps in determining whether hypotheses can be supported or not, and the level is ≤0.05.

## 4 DISCUSSION AND IMPLICATIONS

The hypotheses testing results on consumers’ attitudes toward 3D food printing and 3D‐printed products reveal several significant predictors. Knowledge awareness (*β* = 0.103, *t*‐value = 0.035, *p*‐value = 0.035) positively influences the attitude toward 3D‐printed food, indicating that greater knowledge awareness enhances consumers’ attitudes positively. Experience awareness (*β* = 0.162, *t*‐value = 4.118, *p*‐value = 0.000) also has a strong positive impact, more than knowledge awareness, underscoring the importance of practical experience in shaping favorable attitudes. These findings are consistent with the studies by Ng et al. (2022) and Brunner et al. (2018), which found that greater knowledge awareness among respondents significantly enhanced their attitude toward 3D‐printed food. This suggests that within an African context, where it may be assumed that consumers lack awareness of 3D‐printed food because of its infancy (Fasogbon & Adebo, 2022), the efforts in creating both knowledge and experience awareness should not be ignored, given their potential impact on attitude.

The perception of benefits (*β* = 0.494, *t*‐value = 9.920, *p*‐value = 0.000) is the most substantial positive influencer, suggesting that perceived benefits (i.e., ease of producing food, personalizing nutrients, safety, reducing food wastage) of 3D‐printed food significantly drive positive attitudes. Some studies have highlighted the positive influence of perceived benefits on consumers’ attitudes toward 3D‐printed food. For instance, Brunner et al. (2018) conducted a study with 260 Switzerland residents aged 18–80, revealing that perceptions of fun, convenience, health, and nutrition are crucial in promoting 3D food printing to early adopters. Similarly, an online survey by Ng et al. (2022) among Malaysian consumers found that perceived benefits significantly contributed to positive attitudes toward 3D‐printed food. This indicates that consumers may question the necessity and advantages of novel food technologies when there is a lack of perceived benefits. Consequently, this supports the importance of perceived benefits from both a theoretical and practical perspective, whereby 3D‐printed food manufacturers would want to stress the perceived benefits associated with consuming 3D‐printed food.

Conversely, food familiarity (*β* = ‐0.004, *t*‐value = 0.935, *p*‐value = 0.935) showed no significant effect, implying that being familiar with the processing method does not affect attitudes toward 3D‐printed food. This contradicts the findings of Lupton and Turner (2018), who asserted that consumers’ familiarity with the technology could enhance their acceptance of 3D‐printed food. It also contradicts the findings of Ng et al. (2022), which indicated that respondents' lack of familiarity with the technology significantly contributed to their negative attitudes toward 3D‐printed food.

Food neophobia (*β* = ‐0.183, *t*‐value = 3.667, *p*‐value = 0.000) was found to negatively impact attitude, indicating that fear of new food hinders acceptance as predicted. Given that 3D food printing is a new and largely unknown technology in South Africa, it is unsurprising that respondents expressed some apprehension toward it. This negative impact of food neophobia on consumers’ attitudes toward 3D‐printed food aligns with findings by other researchers (Brunner et al., 2018; Ng et al., 2022). Furthermore, the finding suggests that consumers are hesitant to try new and different food that requires further education about the benefits that 3D‐printed food can offer consumers, such as health. Health (*β* = 0.109, *t*‐value = 2.223, *p*‐value = 0.026) was found to have a positive and significant impact on the attitude toward 3D‐printed food. This indicates that informing consumers about the health benefits of 3D‐printed food can positively influence their attitude toward this emerging technology. This is in agreement with the findings of Silva et al. (2024), which showed that respondents exhibited a positive attitude toward 3D‐printed food after learning about its potential health benefits, in contrast to their initial skepticism when no health benefits were mentioned. This provides support for the health benefits to be at the forefront of the marketing strategies behind 3D‐printed food to encourage consumers’ attitudes. According to Baiano (2022), the health benefits relating to 3D‐printed food are endless, as the food is being enhanced with the required nutrients for specific groups of individuals, such as a snack developed for children aged 3–10 whereby specific nutrients (energy, calcium, iron, and vitamin D) were incorporated into the food.

The convenience orientation (*β* = 0.075, *t*‐value = 1.345, *p*‐value = 0.179) was found not to be a significant factor, suggesting that the convenience aspect does not substantially affect attitudes toward 3D‐printed food. This contradicts the findings of Brunner et al. (2018), who found convenience to have a significant and positive impact on the attitude toward 3D‐printed food. This could be linked to the finding relating to food familiarity—as South African consumers are unfamiliar with 3D‐printed food, they are unable to determine the convenience associated with 3D‐printed food. Therefore, consumers are not driven to form positive attitudes toward 3D‐printed food due to their lack of convenience orientation.

## 5 LIMITATIONS OF THE STUDY

The study used a limited number of respondents (355), though broadly covering different age groups, genders, and social strata. This is not unusual when compared with some previous similar studies, including that of Manstan and McSweeney (2020), which reported the use of 329 respondents. Consequently, the study's findings are limited to the responses from these groups of individuals. In addition, the study was conducted within a country where 3D‐printed food is still in its infancy, which may have had an unintended impact on the research findings. Lastly, although the quantitative nature of the study presents unique insights into consumers’ attitudes toward 3D‐printed food, the quantitative nature of the study does not provide an in‐depth understanding of consumers’ attitudes, such as why they are unfamiliar with 3D‐printed food and do not regard it as a convenient method.

## 6 CONCLUSION AND FUTURE RESEARCH

This study provided valuable insights into consumer attitudes toward 3D‐printed food within the African context, where the technology is in the infancy stage. Overall, the study highlights that while knowledge, experience, perceived benefits, and health considerations positively shape consumers’ attitudes toward 3D‐printed food, food familiarity and convenience orientation do not play significant roles, and food neophobia poses a barrier. This suggests that consumers regard the benefits of 3D‐printed food as the most significant driver toward forming their positive attitudes, which signals the importance of focusing on the benefits when promoting 3D‐printed food. In addition, the marketers and manufacturers of 3D‐printed food and devices, as well as government entities, should ensure that they focus on educating consumers on the food and the way in which the technology can enhance the nutrient value. As food neophobia was shown to have a significant negative relationship with attitude, this poses a substantial threat to the adoption of 3D‐printed food.

Future research should investigate the underlying causes of neophobia toward 3D‐printed food, distinguishing whether it stems from the food itself or the technology used. Additionally, studies should examine consumer age differences to determine if younger consumers exhibit more positive attitudes due to greater technological exposure. Research on consumer perceptions of different types of 3D‐printed food (e.g., plant‐based vs. animal‐based) could also provide valuable insights. Finally, cross‐country comparative studies in regions where 3D‐printed food is more established may yield unique findings.

## AUTHOR CONTRIBUTIONS

**Nicole Cunningham**: Methodology; visualization; investigation; writing—original draft; and writing—review & editing. **Adeyemi Ayotunde Adeyanju**: Validation; visualization; writing—review & editing; writing—original draft. **Oluwafemi Ayodeji Adebo**: Writing—review & editing; writing—original draft; funding acquisition; project administration; visualization; validation; methodology; conceptualization.

## CONFLICT OF INTEREST STATEMENT

The authors declare no conflict of interest.
